# Educational and Social Exergaming: A Perspective on Physical, Social, and Educational Benefits and Pitfalls of Exergaming at Home During the COVID-19 Pandemic and Afterwards

**DOI:** 10.3389/fpsyg.2021.644036

**Published:** 2021-04-09

**Authors:** Marco Rüth, Kai Kaspar

**Affiliations:** Department of Psychology, University of Cologne, Cologne, Germany

**Keywords:** COVID-19, exergaming, physical activity, social gaming, social learning, media education, home exercising

## Abstract

Physical inactivity and coronavirus disease 2019 (COVID-19) signify two pandemics with negative physical, mental, and economic consequences. Younger and older people have not reached the recommended physical activity level for years. Societal restrictions due to COVID-19 additionally reduce opportunities for physical activity, and they increase social isolation. Here, we outline how playing exergames with others (social exergaming) at home could foster physical and mental health and promote communication and discussions on exergaming. Accordingly, we highlight the educational and social benefits of exergaming at home and delineate the concept of Educational and Social Exergaming (EASE). We outline specific benefits and pitfalls of exergaming regarding its physical and nonphysical effects, including educational values of discussing exergaming experiences and related topics. Moreover, we discuss the relevance of practical guidelines for educational and social exergaming at home as well as prospects for future research. Overall, educational and social exergaming could alleviate several detrimental effects of both pandemics on the health and well-being of people of all ages.

## Introduction

Physical activity and exercising can promote health and well-being for people of all ages, but worldwide, recommended levels of physical activity are not reached by about 80% of adolescents (Guthold et al., 2020) and by almost 30% of adults (Guthold et al., 2018). This problem is exacerbated by restrictions on daily life and on physical activity associated with the coronavirus disease 2019 (COVID-19) pandemic (López-Bueno et al., 2020; Rhodes et al., 2020; Santos et al., 2021). Positive associations were found between physical activity and participation in community sports, access to sports and recreational facilities, and time outdoors (Sterdt et al., 2014). However, pandemic restrictions reduce health benefits that sport club activities (Kokko et al., 2019) and leisure-time physical activity (Saint-Maurice et al., 2019) could generate. Importantly, lower levels of physical activity may also be more likely to lead to social isolation (Herbolsheimer et al., 2018; Werneck et al., 2019). Overall, physical inactivity and COVID-19 signify two different but partly intertwined pandemics with negative physical, mental, and economic consequences that must be alleviated (Ding et al., 2016; Hall et al., 2020).

Active video gaming (exergaming) appears to be a suitable option to stay physically active at home, for instance, with balance training, dancing, boxing, and tennis (Street et al., 2017). Several exergames mean one-time investments, can be used with available devices (e.g., smartphones and an internet-connected screen), and provide social connections to many players (e.g., gaming communities, Goodman et al., 2018). Developers of exergames can quickly adapt to changes such as the current pandemic (cf. Laato et al., 2020). Nevertheless, there are also long-lasting exergame series, for instance, dance exergames that can be played on different consoles or only with a smartphone and a display device (e.g., Ubisoft, 2021a,b). Hence, the gaming market is constantly changing, and new applications and technical devices may also emerge from innovative research.

Not surprisingly, exergaming has also been suggested as one promising way of home exercising during the current pandemic (e.g., Chtourou et al., 2020). Previous research also outlined several strengths and limitations of using exergames by people of all ages (Stanmore et al., 2017; Kappen et al., 2019; O'Loughlin et al., 2020). However, in contrast to scientific studies using controlled exergaming settings, the realization of exergaming at home leaves much room for success and failure. The present conceptual work, hence, outlines a detailed perspective on potential benefits and pitfalls.

## The Meaning and Value of Educational and Social Exergaming at Home

**Figure 1:**
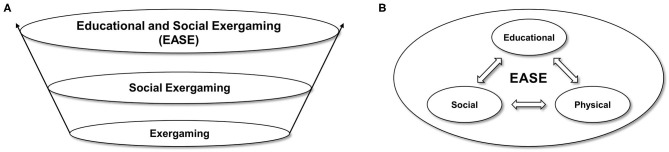
Educational and Social Exergaming (EASE). **(A)** The pure physical dimension, which is addressed by exergaming by definition, is supplemented by the social dimension of social exergaming and by an educational dimension, forming three levels. The highest level is represented by EASE. However, the vertical transitions between the model's levels are fluid rather than categorical. **(B)** The physical, social, and educational dimensions of EASE are strongly interrelated.

Playing exergames with others (social exergaming) can be realized locally within a household or online via the internet. Social exergaming can not only foster physical activity but also provide social support and create valuable opportunities to communicate with others (Marker and Staiano, 2015). Indeed, social interactions are a key element of physical activity (Best et al., 2017) and some video games (Rogers, 2017). Hence, social exergaming might help to counteract negative nonphysical impacts of pandemic restrictions, such as social isolation (Bentlage et al., 2020) or anxiety (Viana and de Lira, 2020). Moreover, the value of exergames for physical education is apparent (e.g., Ennis, 2013; Vaghetti et al., 2018), yet we highlight that exergaming experiences can foster more general media competences that have been widely neglected so far in the context of exergames. Hence, social exergaming can foster physical and mental health, and it provides experiences being worth to be reflected from an educational perspective, forming the concept of *Educational and Social Exergaming (EASE)* (see Figure 1). Accordingly, we outline how physical, social, and educational aspects can contribute to a successful and satisfying exergaming experience at home and how these aspects are interrelated.

### Physical Effects of Exergaming at Home

Physical effects of exergaming include (neuro-)plasticity and motor learning that take place via (neuro-)biological, physiological, and other mechanisms (e.g., Sigrist et al., 2013; Voss et al., 2013; Lubans et al., 2016). As summarized by Table 1, exergaming can unfold several positive and negative effects on the physical dimension. Importantly, meta-analytic results indicate that exergaming can lead to substantially higher physical activity than sedentary behavior (Santos et al., 2021). In addition, strong correlations between exergaming and increases in energy expenditure were reported in several studies (Sween et al., 2014). During a pandemic, exergames can assist in establishing or maintaining structured and regular activity routines in (small) home environments (Chtourou et al., 2020). Exergaming can also complement or even (temporarily) substitute other physical activities (Street et al., 2017) and support therapeutic and rehabilitative procedures in home environments (Ambrosino et al., 2020). However, a meta-analysis focusing on home and lab environments (Oliveira et al., 2020) revealed that exergaming was better in reducing the body mass index but not in increasing physical activity levels in young people, compared to minimal interventions, such as education, sedentary video gaming, or waiting. These effects were similar at short term (<3 months) and intermediate term (3–12 months). In their review, Baranowski et al. (2014) also reported mixed results regarding the effect of exergaming interventions on physical activity; the authors highlighted that interventions could be more effective if they include instructions or are embedded in a structured program. Besides methodological issues questioning some of the previously reported results, effective exergaming regarding the physical domain, hence, seems to depend on several moderating factors.

**Table 1:** Potential benefits and pitfalls of educational and social exergaming at home.

	**Potential benefits: Exergaming…**	**Potential pitfalls: Exergaming…**
Physical effects of exergaming at home	…stimulates adequate and sufficient physical activity and energy expenditure	…promotes inappropriate and insufficient physical activity and energy expenditure
	…establishes or maintains structured and regular activity routines in (small) home environments	…takes place in unstructured and erratic ways (e.g., since no instructions are provided)
	…complements or even (temporarily) substitutes other physical activities	…is an ineffective complementation or substitution of other physical activities
	…supports therapeutic and rehabilitative procedures in home environments	…does not (appropriately) consider specific needs of users
	…is enjoyable and motivates to engage in physical activity	…does not motivate for physical activity due to suboptimal procedures and features
	…includes real-time and informative (formative) feedback on users' performance	…does not include adequate, preferred, or professional feedback
	…allows to engage in physical activity at a customizable training intensity	…results in unhealthy exercising due to inappropriate training intensity
	…allows to assess the goal attainment level and includes (summative) feedback	…is aimless (e.g., no learning objectives and no specific goals are provided prior to exergaming)
Nonphysical effects of social exergaming at home	…in competitive and/or cooperative modes elicits effects on a motivational, emotional, and cognitive level that foster physical activity (e.g., higher self-efficacy and physical effort)	…in competitive and/or cooperative modes elicits effects on a motivational, emotional, and cognitive level that hamper physical activity or cause undesirable side effects (e.g., physical aggression)
	…is motivating with suitable teammates or opponents due to higher perceived relatedness	…does not promote relatedness; there is even a risk of social exclusion in competition modes
	…means a social family activity that fosters family satisfaction and family closeness	…is inadequately integrated into daily life; competition modes might stress family harmony
	…provides motivating and feasible challenges that improve (perceived) competences	…provides frustrating and unfeasible challenges that counteract competence acquisition
	…is motivating, as there is an appropriate level of voluntariness and choice (high autonomy)	…is not motivating because it is forced or because it offers little control (low autonomy)
	…is motivating since it offers new experiences (high novelty).	…is not motivating since it is monotonous (low novelty).
	…is motivating due to social support from family, friends, and (significant) others	…lacks (adequate) social support so that exergaming might not be initiated or retained
Educational values of discussing exergaming and related topics at home	…provides shared and discussable experiences (e.g., physical and nonphysical effects)	…provides experiences that are not shared or not considered worthy to be discussed
	…facilitates discussing media-driven topics (e.g., exergames' features, ubiquity of technology)	…is used but media-driven topics are not (adequately) discussed (e.g., due to insufficient knowledge about media)
	…facilitates discussing education-driven topics (e.g., digital competences, mediation styles)	…is used but education-driven topics are not (adequately) discussed (e.g., due to insufficient digital competences)
	…facilitates discussing health-related topics (e.g., loneliness, behavioral addictions)	…is used but health-related topics are not (adequately) discussed (e.g., due to insufficient health-related knowledge)
	…offers opportunities for (intergenerational) communication that can alleviate loneliness and social anxiety and allow learning with others	…offers little or no opportunity for communication; discussing results in conflicts with others or the termination of exergaming

Exergaming may be preferred over other physical activities due to its game-oriented characteristics. Enjoyment is commonly experienced during video gaming (Ryan et al., 2006), and it is the key to motivate for physical activity (Lewis et al., 2016; Best et al., 2017). Therefore, procedures and features that enhance enjoyment in exergames are important and heavily discussed, such as aesthetical aspects, game mechanics, and story elements (e.g., Mellecker et al., 2013; Baranowski et al., 2014). An effective promotion of physical activity also requires real-time and informative (formative) feedback about users' performance, a customizable training intensity, and an assessment of and (summative) feedback on the goal attainment level (cf. Sigrist et al., 2013). Importantly, users' preference for a specific feedback type may contradict its objective effectiveness (Greinacher et al., 2020). In general, feedback is essential for motor learning (Sigrist et al., 2013; Wulf, 2013), also because the variability that humans show in their movements seems to be important for motor learning (Dhawale et al., 2017). In addition, positive effects on body composition and physical activity may disappear given no learning objectives and no specific goals prior to exergaming (Gao et al., 2020). Moreover, several nonphysical processes can affect physical activity specifically when it comes to social exergaming.

### Nonphysical Effects of Social Exergaming at Home

Exergaming can elicit several nonphysical (psychological) effects on the motivational, emotional, and cognitive level in clinical and nonclinical populations (for reviews, see Joronen et al., 2017; Lee et al., 2017; Stanmore et al., 2017; Santos et al., 2021). Prominent theoretical accounts to explain psychological effects and correlates of social exergaming include self-determination theory, social cognitive theory, theory of planned behavior, and social identity theory (e.g., Nasuti and Rhodes, 2013; Marker and Staiano, 2015). Here, we emphasize potential benefits and pitfalls that can be attributed to the social dimension of exergaming (see Table 1).

Social exergaming principally means to play against (competitively) and/or with (cooperatively) other players. Compared to exergaming alone, competitive exergaming against peers was found to reduce perceived exertion and to increase affective outcomes of children and adolescents (Lisón et al., 2015). Moreover, competitive exergaming could increase physical effort or even aggression when exergames contain violent behavior (Marker and Staiano, 2015). Cooperative exergaming can foster motivation, game continuance, and self-efficacy as well as increase prosocial behaviors, for instance, when parents play with their children (Marker and Staiano, 2015). Exergames also allow for cooperative competitions: In a school environment, students in sixth grade danced in groups and competed against each other. The students reported not only high dance and game enjoyment across 4 weeks but also considerable group cohesion in terms of social attraction to group members (Rüth and Kaspar, 2020). Given these results, social exergaming seems recommendable over exergaming alone in some cases, yet more social factors come into play.

Relatedness (to engage with others) is a basic human need besides competence (to perceive skill increase) and autonomy (to endorse activities oneself) in terms of self-determination theory (Standage and Ryan, 2020). First, users can experience higher social relatedness when playing exergames online with peers compared to playing against a computer opponent (Kooiman and Sheehan, 2015). However, in competition modes, there is a general risk of social exclusion processes as indicated by research on (minimal) group membership (cf. Lelieveld et al., 2020). In turn, exergaming experiences can be more motivating and satisfactory given suitable teammates or opponents (cf. Chan et al., 2019). Relatedly, families that video gamed together reported better family satisfaction and closer relationships within the family (Wang et al., 2018). Second, perceived competence was found to be lower when exergaming compared to exercising (Osorio et al., 2012). In general, task difficulty should be adapted to players' skill level and competences as well as their individual learning curve (Kiili, 2005; Hardy et al., 2015). Third, children who were offered a high level of autonomy in terms of a free choice to play exergames or sedentary video game alternatives played both types of games for a similar amount of time (Lam et al., 2011). Notably, social exergaming could also satisfy a (fourth) basic need for novelty (Vansteenkiste et al., 2020). While offering new experiences had a positive indirect effect on the intention to be physically active via autonomous motivation in school-aged students (Fernández-Espínola et al., 2020), playing with other players can result in new experiences. However, novelty effects (a rapid decline from an initially high level of appeal or usage) might also lead to an overestimation of exergames' (long-term) effects. Overall, the fulfillment of basic human needs fosters motivation, well-being, and personal growth.

Social exergaming can be further enriched via social support, which means that people exchange various kinds of social resources to improve their mental well-being (cf. Zimet et al., 1988). Examples of social support include emotional support (e.g., parents who care for their children) or exchange of information (e.g., friends who give advice). For children and adolescents, social support of parents, friends, and significant others was found to positively correlate with physical activity (Sterdt et al., 2014), whereas meta-analytic results indicate small effects of parental support on physical activity (Yao and Rhodes, 2015). Still, more recently, parental support for physical activity was found to be a key predictor of their children's physical activity (Best et al., 2017). Social support from family members also seems to specifically benefit older adults' physical activity (Smith et al., 2017). In families, parents can support the initiation of exergaming, and siblings can support adherence (Baranowski et al., 2014). Previous research also reported positive associations between social support from friends and physical activity (Hamilton et al., 2017; Scarapicchia et al., 2017). Nonetheless, further research on social support during exergaming is needed (Gao et al., 2020). Conversations about exergaming could also provide social support and needs support, for instance, of relatedness by showing authentic interest in a person (Standage and Ryan, 2020). Consequently, social exergaming has the potential to help people cope with pandemic restrictions on social contacts.

### Educational Values of Discussing Exergaming and Related Topics at Home

Communication can alleviate loneliness and social anxiety (Bonetti et al., 2010; Chipps et al., 2017). With limited access to formal education (UNESCO, 2021), particularly children and adolescents more likely feel lonely without participation in physical education (Pinto et al., 2019). We argue that exergaming provides valuable opportunities to reflect on the meanings of media as environments (media-driven) and the promotion of critical thinking and digital skills (education-driven), which is important to people of all ages (Rasi et al., 2019). Therefore, EASE at home could provide educational values through discussions that can also promote media competences (see Table 1). We present four possible (intertwined) directions for joint discussions at home.

First, players' shared exergaming experiences (e.g., successes or failures) and related physical and nonphysical effects could serve as obvious conversation starters. Players could reflect on their physical activity, including motor and cognitive skills, values of physical activity, and motivational and social aspects (Corbin, 2016). Even before exergaming, parents could explain their children what it means to participate in competitive vs. cooperative exergaming. Discussing game experiences offers opportunities to practice media criticism, and this process can be supported by parents or teachers (Rüth and Kaspar, 2021).

Second, media-driven topics include understanding the exergames' features such as the motion sensors, which are often readily accessible in the form of visual, performance-based feedback. This could also facilitate a visual approach to complex topics such as the ubiquity of technology and the associated generation, measurement, and surveillance of data in daily life (Mascheroni, 2020).

Third, education-driven topics include diverse aspects of media education that require standards (Blumberg et al., 2019) and prioritization (Fedorov et al., 2016). Still, available frameworks allow examinations of key digital competence areas at different proficiency levels (Carretero et al., 2017). Further, parents usually regulate how their children use video games (ESA, 2020). Accordingly, mediation styles, such as restrictive use (limited time or content) or co-use (playing together) (Lorenz and Kapella, 2020), could be discussed (e.g., why parents allow exergaming and restrict sedentary video gaming).

Fourth, video games and digital technology are prevalent in peoples' daily lives, even more so during COVID-19 pandemic restrictions (GWI, 2020; Newzoo, 2020). Relatedly, excessive sedentary screen time can hamper physical and mental health (Twenge and Campbell, 2018; Engberg et al., 2019), and excessive online communication can increase loneliness (Boursier et al., 2020). Therefore, potential unhealthy consequences could be discussed and related to exergaming, including addictions regarding video gaming (Paulus et al., 2018; WHO, 2020a) and the internet (Venkatesh et al., 2019; Dong et al., 2020).

Overall, many more directions are conceivable, but who is talking to whom at all? Communicators can be of different ages and include (grand)parents and their (grand)children, siblings, friends, and players from online communities. When older people play with younger people, it could also improve intergenerational communication (Costa and Veloso, 2016) and decrease social anxiety in older people (Xu et al., 2016). Moreover, communicators could increase their interpersonal competences, such as providing emotional support, and thereby reduce their stress and loneliness (Segrin, 2019). Communicators can influence each other and learn from each other cooperatively (cf. Butera and Buchs, 2019). While teacher-led discussions can support students to reflect on video game experiences in formal school teaching (Rüth and Kaspar, 2021), question catalogs and interview guidelines could facilitate and structure communications in informal learning environments at home. That said, care should be taken to balance exergaming with serious discussions so that players enjoy and engage in exergaming also in the long run.

## Discussion

After outlining several *potential* benefits and pitfalls related to the concept of EASE, we now turn to its feasibility. Exergaming interventions should provide appropriate levels of physical activity, enjoyment, and adherence but low additional costs (LeBlanc et al., 2013). First, one needs to select and use exergames appropriately (considering exercise frequency, intensity, timing, type, and context) to promote health benefits and to avoid negative effects on the immune system (Lubans et al., 2016; Chtourou et al., 2020). Existing guidelines for physical activity (e.g., Dwyer et al., 2020; WHO, 2020b) or for the parental control of video game use (e.g., ESRB, 2021; ISFE, 2021) can provide some orientation. Second, people could adhere to exergaming if it satisfies their basic needs. Techniques such as self-monitoring, self-reinforcement, or motivational interviewing can also promote long-term play (cf. Hardcastle et al., 2015). Third, additional costs include time, budget, and adverse events. For instance, integrating game-like elements into common activities (e.g., rewards, such as points or badges for rope skipping, Fang et al., 2019) has low additional costs and provides new experiences, even if suitable exergames are not available or affordable. Overall, recommendations depend on several situational factors.

EASE at home may benefit from structured programs and professional guidance. Therapeutic and rehabilitative applications would even require professional support (Ambrosino et al., 2020). Online video communication allows coaches to provide professional feedback (cf. Chtourou et al., 2020) and to improve physical activity and weight-related outcomes (Staiano et al., 2018). Supportive parent–child communication can also alleviate negative effects of children's digital technology use on their life satisfaction (Boniel-Nissim et al., 2015) and their risk of becoming a victim of cyberbullying (Buelga et al., 2017). Moreover, child abuse potential was found to be higher when parents were more stressed due to feelings of anxiety and depression related to COVID-19 but lower when parents provided parental support (Brown et al., 2020). Nevertheless, a successful promotion of exergaming and physical activity at home is needed (cf. Ainsworth and Ananian, 2020; Williamson et al., 2020). Taken together, structured programs and professional guidance could be important cornerstones for effective implementations of EASE, yet more high-quality evidence on EASE is needed.

There is a lack of research on exergaming in home environments, and the fidelity of interventions should be ensured (Gao et al., 2020). Moreover, heterogeneity in the exergaming literature complicates generalizability and comparability of evidence and hampers theoretical and practical progress (O'Loughlin et al., 2020). While heterogeneity partly relies on situational factors, some convergence could result from evaluating sound theoretical frameworks such as self-determination theory (Standage and Ryan, 2020). Several suggestions for future research on exergaming (e.g., Straker et al., 2015; Baranowski et al., 2016; O'Loughlin et al., 2020) and general guidelines for media-based interventions (cf. Rüth and Kaspar, 2017) should also be considered when investigating home environments. In the Supplementary Material, we outline some avenues for future research on EASE regarding research approaches, measurement of dependent variables, and effects of exergaming elements. In the long term, databases might help to converge evidence across contexts and to determine at-risk groups (cf. Ayllón et al., 2020), in accordance with respective laws on data protection and appropriate ethical standards. Overall, we recommend the development and use of evidence-based guidelines for (investigations of) exergaming at home.

To conclude, EASE can alleviate detrimental effects of pandemic restrictions on physical and mental health and foster media competences in home environments. While the COVID-19 pandemic has severe consequences on daily lives and physical activity, its end will not put an end to the pandemic of physical inactivity. Therefore, this work is not limited to the current COVID-19 restrictions.

## Data Availability Statement

The original contributions presented in the study are included in the article/Supplementary Material, further inquiries can be directed to the corresponding author.

## Author Contributions

MR and KK conceptualized the manuscript. MR drafted and revised the manuscript. KK revised the manuscript. All authors contributed to the article and approved the submitted version.

## Conflict of Interest

The authors declare that the research was conducted in the absence of any commercial or financial relationships that could be construed as a potential conflict of interest.
