# Supplementary material for: Educational and Social Exergaming: A Perspective on Physical, Social, and Educational Benefits and Pitfalls of Exergaming at Home During the COVID-19 Pandemic and Afterwards
Source: Front Psychol. 2021 Apr 9;12:644036. doi: 10.3389/fpsyg.2021.644036 (PMC8062880; doi:10.3389/fpsyg.2021.644036)
Supplement: Supplementary file 1 [file Data_Sheet_1.pdf]

Supplementary Materials

With reference to our concept of Educational and Social Exergaming (EASE) at home, we would like to suggest several directions for future research. All directions refer to previous conceptual and empirical works. We focus on possible research approaches (Table S1), the measurement of dependent variables (Table S2), and effects of exergaming elements (Table S3).

Supplementary Table S1. Directions for future research on Educational and Social Exergaming (EASE) at home, with a focus on possible research approaches.

Category	Possible directions and related exemplary references
**Examinations of exergaming interventions at home aimed at…**	…supporting physical and mental health (Gao et al., 2020; Santos et al., 2021), for instance, via community-based, family-based, and school-based programs (Christison and Khan, 2012; Baranowski et al., 2014; Rhodes et al., 2018)
	…relatedness and social support, for instance, by connecting players to other players (Chan et al., 2019), e-sports teams/leagues (Martin-Niedecken and Schättin, 2020), and gaming communities in general (Goodman et al., 2018)
	…promoting communication and media competences such as interpersonal competences and cooperative learning (Butera and Buchs, 2019; Segrin, 2019)
	…specific groups of people, such as children with special needs (Flynn and Colon, 2016), people with mobility impairments (Malone et al., 2019), and athletes performing high intensity interval training (Martin-Niedecken et al., 2020; Sá Filho et al., 2020)
	…therapeutic and rehabilitative applications (Ambrosino et al., 2020), including participants with chronic respiratory diseases (Simmich et al., 2019)
	…short- and long-term effects, with a focus on longitudinal studies (Gao et al., 2020; O'Loughlin, Dutczak et al., 2020; Standage and Ryan, 2020)
	…the evaluation and development of theoretical frameworks related to the abovementioned exergaming contexts. Critical conditions supporting theory development have been formulated (e.g., Rüth and Kaspar, 2017)
**Examination of contrasts in exergaming contexts such as…**	…exergaming types (e.g., dance games, sports games etc.) (Oliveira et al., 2020)
	…exergaming modes (e.g., single- and multiplayer settings with cooperation, competition, or coopetition) (Oliveira et al., 2020)
	…exergaming systems (e.g., augmented reality and virtual reality) (Ng et al., 2019)
	…exergaming activities (e.g., balance training, dancing, boxing, and tennis) (Street et al., 2017)
	…exergaming movements (e.g., whole body vs. lower body) (Gao et al., 2015)
	…exergaming contexts, for instance, same vs. other physical space (Peng and Crouse, 2013) or home-based vs. lab-based vs. field-based (Gao et al., 2015; Baranowski et al., 2016; Gao et al., 2020; Oliveira et al., 2020)
	…exergaming vs. other technologies, for instance, gamified apps or wearables (Yen and Chiu, 2019; Schmidt-Kraepelin et al., 2020), home exercising (treadmill, exercise bike, weights/resistance band etc.), and exercise programs (exercise apps, work out videos, virtual personal trainer, virtual workout groups etc.) (Rhodes et al., 2020)
**Examination of moderators in exergaming contexts such as…**	…predictors of physical activity (e.g., peer-modeling and physical self-concept, measured physical fitness, and variables related to social status) (Schmidt et al., 2019)
	…age (to consider developmental stages), culture (Baranowski et al., 2016), and other individual differences; for instance, competitive exergaming might not be the best choice to motivate people with low competitiveness (Song et al., 2013)
	…socioeconomic status and weight status (O'Loughlin, Dutczak et al., 2020)
	…gender, as outcomes for girls and boys may differ (Andrade et al., 2020)
	…group members' performance, as this could affect the effort individuals invest in the activity (Lee et al., 2017)

Supplementary Table S2. Directions for future research on Educational and Social Exergaming (EASE) at home, with a focus on the measurement of dependent variables.

Category	Possible directions and related exemplary references
**Application of instruments regarding physical effects such as…**	…physical activity levels: low physical activity, moderate to vigorous physical activity, and vigorous physical activity (Gao et al., 2015; Oliveira et al., 2020)
	…exercise-related outcomes: energy expenditure, heart rate, metabolic equivalent, perceived exertion, oxygen consumption, body composition, cardiovascular fitness (Gao et al., 2015), and step count (Oliveira et al., 2020)
	…weight-related outcomes: body weight, body fat, waist circumference, (non-)standardized body mass index (Oliveira et al., 2020)
	…wristbands, smartwatches, or smartphones with mobile apps (Yen and Chiu, 2019) or accelerometers (Hwang et al., 2018)
	…engagement in other forms of physical activity due to exergaming (transfer effects); for instance, whether exergaming only motivates people who already do frequently exercise to engage in other forms of exercise (Van Nguyen et al., 2017)
**Application of instruments regarding non-physical effects such as…**	…the Physical Activity Enjoyment Scale (PACES) (Moore et al., 2009)
	…the Basic Psychological Needs in Exercise Scale (BPNES) (Fernández-Espínola et al., 2020)
	…social support scales (e.g., Zimet et al., 1988; Reimers et al., 2012)
	…the Reasons to Exergame (RTEX) scale (O'Loughlin, Sabiston, et al., 2020) and the Motivation for Exergame Play Inventory (MEPI) (Staiano et al., 2019)
	…game experience scales, such as the Player Experience of Need Satisfaction (PENS) and the Game Experience Questionnaire (GEQ) (Johnson et al., 2018), and the Player Experience Inventory (PXI) (Abeele et al., 2020)
	…scales for behavioral addictions (e.g., gaming disorder scales for adolescents, Paschke et al., 2020)
	…several adverse outcomes, including violence, sexual permissiveness, and breach of privacy/confidentiality (Baranowski et al., 2016) or injuries and reduced sleep (O'Loughlin, Dutczak et al., 2020)
**Application of measurements to assess educational outcomes such as…**	…objective tests of knowledge and skills (e.g., Rey et al., 2020)
	…qualitative methods to investigate discussions (e.g., De Vet et al., 2012; Barnett et al., 2014)
	…mixed methods regarding exergaming (e.g., Rüth and Kaspar, 2020) or regarding video game discussions (Rüth and Kaspar, 2021)
**Application of instruments of game-related outcomes such as…**	…pre-game: previous experiences/skills, attitudes, behavioral intentions; for instance, via interviews, questionnaires etc. (cf. Mayer et al., 2014)
	…in-game: game performance (e.g., scores), game play (e.g., effort), game experience (e.g., flow); for instance, via questionnaires or data mining (cf. Mayer et al., 2014)
	…post-game: game experience (e.g., enjoyment), facets of satisfaction (e.g., attractiveness), and learning outcomes (e.g., skills); for instance, via interviews, questionnaires, data mining etc. (cf. Mayer et al., 2014)
**Development and validation of measurement instruments…**	…since validated measurement scales are partially missing for specific contexts (cf. Lee et al., 2017)
	…since existing instruments need further investigations (Hulteen et al., 2020) and may have different psychometric properties in different languages (Sattler et al., 2020)

Supplementary Table S3. Directions for future research on Educational and Social Exergaming (EASE) at home, with a focus on effects of exergaming elements.

Category	Possible directions and related exemplary references
**Effects of basic exergaming elements such as…**	…accurate, reliable, user-friendly, and affordable sensors (Baranowski et al., 2014)<br><br>…game design elements, including interactivity, feedback, control, identity, immersion, and storytelling (Baranowski et al., 2016)<br><br>…adaptive game mechanisms such as personalized difficulty to increase flow (Chan et al., 2019)<br><br>…appropriate matchmaking strategies to increase social connectedness (Chan et al., 2019)
**Effects of theory-related exergaming features such as…**	…fulfilment of basic human needs (autonomy, competence, relatedness, and novelty) (cf. Fernández-Espínola et al., 2020; Vansteenkiste et al., 2020). For instance, game features that were expected to satisfy autonomy and competence needs in young adults were found to have small effects (Peng et al., 2012)
**Effects of evidence-based design recommendations such as…**	…regarding competence: provide feedback, challenge, and rewards (Chan et al., 2019)<br><br>…regarding narrative transportation: use of game characters, considering ethnic similarity between game characters and players (Chan et al., 2019)<br><br>…regarding relevance: use characters that players can self-identify with (Chan et al., 2019)
**Effects of best practice principles such as…**	…for different phases: acquisition, learning, and habitual use of exergaming (cf. Straker et al., 2015)<br><br>…implementing health considerations in exergames, which were found to predict the exergaming behavior of college students (Su and Zeng, 2020)

References

Abeele, V. V., Spiel, K., Nacke, L., Johnson, D., and Gerling, K. (2020). Development and validation of the player experience inventory: A scale to measure player experiences at the level of functional and psychosocial consequences. *International Journal of Human-Computer Studies, 135,* 102370. doi: 10.1016/j.ijhcs.2019.102370

Ambrosino, P., Fuschillo, S., Papa, A., Di Minno, M. N. D., and Maniscalco, M. (2020). Exergaming as a supportive tool for home-based rehabilitation in the COVID-19 pandemic era. *Games for Health Journal*, *9,* 311–313. doi: 10.1089/g4h.2020.0095

Andrade, A., Cruz, W. M. D., Correia, C. K., Santos, A. L. G., and Bevilacqua, G. G. (2020). Effect of practice exergames on the mood states and self-esteem of elementary school boys and girls during physical education classes: a cluster-randomized controlled natural experiment. *PLOS ONE*, *15*, e0232392. doi: 10.1371/journal.pone.0232392

Baranowski, T., Maddison, R., Maloney, A., Medina Jr, E., and Simons, M. (2014). Building a better mousetrap (exergame) to increase youth physical activity. *Games for Health Journal, 3*, 72–78. doi: 10.1089/g4h.2014.0018

Baranowski, T., Blumberg, F., Buday, R., DeSmet, A., Fiellin, L. E., ... and Young, K. (2016). Games for health for children – current status and needed research. *Games for Health Journal*, *5,* 1–12. doi: 10.1089/g4h.2015.0026

Barnett, L. M., Ridgers, N. D., Hanna, L., and Salmon, J. (2014). Parents' and children's views on whether active video games are a substitute for the 'real thing'. *Qualitative Research in Sport, Exercise and Health*, *6*, 366–381. doi: 10.1080/2159676X.2013.809379

Butera, F., and Buchs, C. (2019). "Social interdependence and the promotion of cooperative learning", in *Social Psychology in Action,* eds K. Sassenberg and M. L. W. Vliek (Cham: Springer), 111–127. doi: 10.1007/978-3-030-13788-5_8

Chan, G., Arya, A., Orji, R., and Zhao, Z. (2019). Motivational strategies and approaches for single and multi-player exergames: A social perspective. *PeerJ Computer Science, 5*:e230. doi: 10.7717/peerj-cs.230

Christison, A., and Khan, H. A. (2012). Exergaming for health: a community-based pediatric weight management program using active video gaming. *Clinical Pediatrics, 51*, 382–388. doi: 10.1177/0009922811429480

De Vet, E., Simons, M., and Wesselman, M. (2012). Dutch children and parents' views on active and non-active video gaming. *Health Promotion International*, *29*, 235–243. doi: 10.1093/heapro/das064

Fernández-Espínola, C., Almagro, B. J., Tamayo-Fajardo, J. A., and Sáenz-López, P. (2020). Complementing the self-determination theory with the need for novelty: motivation and intention to be physically active in physical education students. *Frontiers in Psychology*, *11*:1535. doi: 10.3389/fpsyg.2020.01535

Flynn, R. M., and Colon, N. (2016). Solitary active videogame play improves executive functioning more than collaborative play for children with special needs. *Games for Health Journal*, *5,* 398–404. doi: 10.1089/g4h.2016.0053

Gao, Z., Chen, S., Pasco, D., and Pope, Z. (2015). A meta-analysis of active video games on health outcomes among children and adolescents. *Obesity Reviews*, *16*, 783–794. doi: 10.1111/obr.12287

Gao, Z., Zeng, N., McDonough, D. J., and Su, X. (2020). A systematic review of active video games on youth's body composition and physical activity. *International Journal of Sports Medicine, 41*, 561–573. doi: 10.1055/a-1152-4959

Goodman, W., McFerran, E., Purves, R., Redpath, I., and Beeken, R. J. (2018). The untapped potential of the gaming community: narrative review. *JMIR Serious Games*, *6*:e10161. doi: 10.2196/10161

Hulteen, R. M., Barnett, L. M., True, L., Lander, N. J., del Pozo Cruz, B., and Lonsdale, C. (2020). Validity and reliability evidence for motor competence assessments in children and adolescents: a systematic review. *Journal of Sports Sciences*, *38*, 1717–1798. doi: 10.1080/02640414.2020.1756674

Hwang, J., Fernandez, A. M., and Lu, A. S. (2018). Application and validation of activity monitors' epoch lengths and placement sites for physical activity assessment in exergaming. *Journal of Clinical Medicine*, *7*, 268. doi: 10.3390/jcm7090268

Johnson, D., Gardner, M. J., and Perry, R. (2018). Validation of two game experience scales: The Player Experience of Need Satisfaction (PENS) and Game Experience Questionnaire (GEQ). *International Journal of Human-Computer Studies*, *118*, 38–46. doi: 10.1016/j.ijhcs.2018.05.003

Lee, S., Kim, W., Park, T., and Peng, W. (2017). The psychological effects of playing exergames: a systematic review. *Cyberpsychology, Behavior, and Social Networking, 20,* 513–532. doi: 10.1089/cyber.2017.0183

Malone, L. A., Thirumalai, M., Padalabalanarayanan, S., Neal, W. N., Bowman, S., and Mehta, T. (2019). Energy expenditure and enjoyment during active video gaming using an adapted Wii Fit balance board in adults with physical disabilities: observational study. *JMIR Serious Games*, *7*:e11326. doi: 10.2196/11326

Martin-Niedecken, A. L., and Schättin, A. (2020). Let the body'n'brain games begin: toward innovative training approaches in eSports athletes. *Frontiers in Psychology, 11*:138. doi: 10.3389/fpsyg.2020.00138

Martin-Niedecken, A. L., Mahrer, A., Rogers, K., de Bruin, E. D., and Schättin, A. (2020). "HIIT" the ExerCube: comparing the effectiveness of functional high-intensity interval training in conventional vs. exergame-based training. *Frontiers in Computer Science, 2*:33. doi: 10.3389/fcomp.2020.00033

Mayer, I., Bekebrede, G., Harteveld, C., Warmelink, H., Zhou, Q., Van Ruijven, T., ... and Wenzler, I. (2014). The research and evaluation of serious games: toward a comprehensive methodology. *British Journal of Educational Technology, 45,* 502–527. doi: 10.1111/bjet.12067

Moore, J. B., Yin, Z., Hanes, J., Duda, J., Gutin, B., and Barbeau, P. (2009). Measuring enjoyment of physical activity in children: validation of the Physical Activity Enjoyment Scale. *Journal of Applied Sport Psychology, 21,* S116–S129. doi: 10.1080/10413200802593612

Ng, Y. L., Ma, F., Ho, F. K., Ip, P., and Fu, K. W. (2019). Effectiveness of virtual and augmented reality-enhanced exercise on physical activity, psychological outcomes, and physical performance: a systematic review and meta-analysis of randomized controlled trials. *Computers in Human Behavior, 99*, 278–291. doi: 10.1016/j.chb.2019.05.026

O'Loughlin, E. K., Dutczak, H., Kakinami, L., Consalvo, M., McGrath, J. J., and Barnett, T. A. (2020). Exergaming in youth and young adults: a narrative overview. *Games for Health Journal, 9*, 314–338. doi: 10.1089/g4h.2019.0008

O'Loughlin, E. K., Sabiston, C. M., Kakinami, L., McGrath, J. J., Consalvo, M., O'Loughlin, J. L., and Barnett, T. A. (2020). Development and validation of the Reasons to Exergame (RTEX) scale in young adults: exploratory factors analysis. *JMIR Serious Games*, *8*:e16261. doi: 10.2196/16261

Oliveira, C. B., Pinto, R. Z., Saraiva, B. T., Tebar, W. R., Delfino, L. D., Franco, M. R., ... and Christofaro, D. G. D. (2020). Effects of active video games on children and adolescents: a systematic review with meta-analysis. *Scandinavian Journal of Medicine & Science in Sports, 30*, 4–12. doi: 10.1111/sms.13539

Paschke, K., Austermann, M. I., and Thomasius, R. (2020). Assessing ICD-11 gaming disorder in adolescent gamers: development and validation of the Gaming Disorder Scale for Adolescents (GADIS-A). *Journal of Clinical Medicine, 9*:993. doi: 10.3390/jcm9040993

Peng, W., and Crouse, J. (2013). Playing in parallel: The effects of multiplayer modes in active video game on motivation and physical exertion. *Cyberpsychology, Behavior, and Social Networking, 16*, 423–427. doi: 10.1089/cyber.2012.0384

Peng, W., Lin, J. H., Pfeiffer, K. A., and Winn, B. (2012). Need satisfaction supportive game features as motivational determinants: an experimental study of a self-determination theory guided exergame. *Media Psychology*, *15*, 175–196. doi: 10.1080/15213269.2012.673850

Reimers, A. K., Jekauc, D., Mess, F., Mewes, N., and Woll, A. (2012). Validity and reliability of a self-report instrument to assess social support and physical environmental correlates of physical activity in adolescents. *BMC Public Health, 12*:705. doi: 10.1186/1471-2458-12-705

Rey, E., Carballo-Fazanes, A., Varela-Casal, C., Abelairas-Gómez, C., on behalf of ALFA-MOV Project collaborators (2020). Reliability of the test of gross motor development: a systematic review. *PLOS ONE, 15*:e0236070. doi: 10.1371/journal.pone.0236070

Rhodes, R. E., Nwachukwu, N., and Quinlan, A. (2018). Family exergaming: correlates and preferences. *Games for Health Journal, 7*, 188–196. doi: 10.1089/g4h.2017.0177

Rhodes, R. E., Liu, S., Lithopoulos, A., Zhang, C.-Q., and Garcia-Barrera, M. A. (2020). Correlates of perceived physical activity transitions during the COVID-19 pandemic among Canadian adults. *Applied Psychology: Health and Well-Being*. doi: 10.1111/aphw.12236

Rüth, M., and Kaspar, K. (2017). The E-Learning Setting Circle: first steps toward theory development in e-learning research. *Electronic Journal of e-Learning, 15*, 94–103.

Rüth, M., and Kaspar, K. (2020). Exergames in formal school teaching: a pre-post longitudinal field study on the effects of a dance game on motor learning, physical enjoyment, and learning motivation. *Entertainment Computing*, *35*:100372. doi: 10.1016/j.entcom.2020.100372

Rüth, M., and Kaspar, K. (2021). Commercial video games in school teaching: two mixed methods case studies on students' reflection processes. *Frontiers in Psychology*, *11*:594013. doi: 10.3389/fpsyg.2020.594013

Sá Filho, A. S., Miranda, T. G., de Paula, C. C., Barsanulfo, S. R., Teixeira, D., Monteiro, D., ... and Machado, S. (2020). COVID-19 and quarantine: expanding understanding of how to stay physically active at home. *Frontiers in Psychology*, *11*:566032. doi: 10.3389/fpsyg.2020.566032

Santos, I. K. D., Medeiros, R. C. D. S. C. D., Medeiros, J. A. D., Almeida-Neto, P. F. D., Sena, D. C. S. D., Cobucci, R. N., ... and Dantas, P. M. S. (2021). Active video games for improving mental health and physical fitness – an alternative for children and adolescents during social isolation: an overview. *International Journal of Environmental Research and Public Health*, *18*:1641. doi: 10.3390/ijerph18041641

Sattler, M. C., Jaunig, J., Tösch, C., Watson, E. D., Mokkink, L. B., Dietz, P., and van Poppel, M. N. (2020). Current evidence of measurement properties of physical activity questionnaires for older adults: an updated systematic review. *Sports Medicine*, *50*, 1271–1315. doi: 10.1007/s40279-020-01268-x

Schmidt, S. C., Schneider, J., Reimers, A. K., Niessner, C., and Woll, A. (2019). Exploratory determined correlates of physical activity in children and adolescents: the MoMo study. *International Journal of Environmental Research and Public Health*, *16*:415. doi: 10.3390/ijerph16030415

Schmidt-Kraepelin, M., Toussaint, P. A., Thiebes, S., Hamari, J., and Sunyaev, A. (2020). Archetypes of gamification: analysis of mHealth apps. *JMIR mHealth and uHealth*, *8*:e19280. doi: 10.2196/19280

Segrin, C. (2019). Indirect effects of social skills on health through stress and loneliness. *Health Communication*, *34*, 118–124. doi: 10.1080/10410236.2017.1384434

Simmich, J., Deacon, A. J., and Russell, T. G. (2019). Active video games for rehabilitation in respiratory conditions: systematic review and meta-analysis. *JMIR Serious Games*, *7*:e10116. doi: 10.2196/10116

Song, H., Kim, J., Tenzek, K. E., and Lee, K. M. (2013). The effects of competition and competitiveness upon intrinsic motivation in exergames. *Computers in Human Behavior*, *29*, 1702–1708. doi: 10.1016/j.chb.2013.01.042

Staiano, A. E., Adams, M. A., and Norman, G. J. (2019). Motivation for exergame play inventory: construct validity and relationship to game play. *Cyberpsychology: Journal of Psychosocial Research on Cyberspace*, *13*:7. doi: 10.5817/CP2019-3-7

Standage, M., and Ryan, R. M. (2020). "Self-determination theory in sport and exercise", in *Handbook of Sport Psychology*, eds G. Tenenbaum and R. C. Eklund (Toronto, Ontario: John Wiley & Sons), 37–56. doi: 10.1002/9781119568124.ch3

Straker, L. M., Fenner, A. A., Howie, E. K., Feltz, D. L., Gray, C. M., Lu, A. S., ... and Barnett, L. M. (2015). Efficient and effective change principles in active videogames. *Games for Health Journal*, *4*, 43–52. doi: 10.1089/g4h.2014.0077

Street, T. D., Lacey, S. J., and Langdon, R. R. (2017). Gaming your way to health: a systematic review of exergaming programs to increase health and exercise behaviors in adults. *Games for Health Journal*, *6*, 136–146. doi: 10.1089/g4h.2016.0102

Su, Z., and Zeng, C. (2020). The effects of health consideration on exergaming behavior in college students: a structural equation perspective. *Research Quarterly for Exercise and Sport*. doi: 10.1080/02701367.2020.1801970

Van Nguyen, H., Huang, H.-C., Wong, M.-K., Lu, J., Huang, W.-F., and Teng, C.-I (2016). Double-edged sword: the effect of exergaming on other forms of exercise; a randomized controlled trial using the self-categorization theory. *Computers in Human Behavior*, *62*, 590–593. doi: 10.1016/j.chb.2016.04.030

Vansteenkiste, M., Ryan, R. M., and Soenens, B. (2020). Basic psychological need theory: advancements, critical themes, and future directions. *Motivation and Emotion*, *44*, 1–31. doi: 10.1007/s11031-019-09818-1

Yen, H. Y., and Chiu, H. L. (2019). The effectiveness of wearable technologies as physical activity interventions in weight control: a systematic review and meta-analysis of randomized controlled trials. *Obesity Reviews*, *20*, 1485–1493. doi: 10.1111/obr.12909
